# Association between physical activity and costs in very mild to moderately frail community-dwelling older adults: a cross-sectional study

**DOI:** 10.1186/s12889-024-20253-x

**Published:** 2024-10-08

**Authors:** Sophie Gottschalk, Hans-Helmut König, Christian Werner, Tim Fleiner, Christian Thiel, Gisela Büchele, Martina Schäufele, Kilian Rapp, Judith Dams

**Affiliations:** 1 Department of Health Economics and Health Services Research, University Medical Center Hamburg-Eppendorf, Hamburg, Germany; 2 Hamburg Center for Health Economics, Hamburg, Germany; 3 Geriatric Centre, Heidelberg University Hospital, Agaplesion Bethanien Hospital Heidelberg, Heidelberg, Germany; 4 Institute for Geriatric Research, Ulm University, Ulm, Germany; 5 Institute of Medical Engineering and Mechatronics, Ulm University of Applied Sciences, Ulm, Germany; 6 Department of Applied Health Sciences, Hochschule für Gesundheit (University of Applied Sciences), Bochum, Germany; 7 Faculty of Sports Science, Ruhr-University Bochum, Bochum, Germany; 8 Institute of Epidemiology and Medical Biometry, Ulm University, Ulm, Germany; 9 Department of Social Work, University of Applied Sciences, Mannheim, Germany; 10 Department of Clinical Gerontology, Robert-Bosch-Hospital, Stuttgart, Germany

**Keywords:** Physical activity, Exercise, Cost, Healthy aging, Frailty

## Abstract

**Background:**

Physical activity (PA) plays a vital role in maintaining the functional ability that enables well-being in older age (healthy aging), potentially also saving costs for the healthcare system and society. The aim of this study was to examine the association between PA and healthcare and societal costs in a sample of very mild to moderately frail older adults.

**Methods:**

This cross-sectional study is a secondary analysis using baseline data from the PromeTheus randomized-controlled trial, which included 385 very mild to moderately frail community-dwelling older adults (70 + years) from Germany. Participants self-reported their health-related resource use in the previous 6 months (FIMA questionnaire), which was monetarily valued using standardized unit costs. PA was also self-reported using the German Physical Activity Questionnaire for middle-aged and older adults (German-PAQ-50+) and categorized as ‘insufficient’/’sufficient’ or ‘insufficient’/‘moderate’/‘high’ in accordance with the World Health Organization guidelines for PA. Mean and median healthcare costs (including outpatient, inpatient, rehabilitation, formal care, and medication costs) and societal costs (healthcare costs plus informal care costs) for different PA groups were estimated using generalized linear models and quantile regression, with sociodemographic variables and physical capacity (Short Physical Performance Battery) as covariates.

**Results:**

Of the sample, 24% were classified as insufficiently, 23% as moderately, and 54% as highly active. Sufficient PA, especially high PA, was associated with lower costs in the 6 months prior to data collection compared to insufficient PA (-€6,237, 95% CI [-10,656; -1,817] and -€8,333, 95% CI [-12,183; -4,483], respectively). The cost difference between PA intensity groups was largely driven by differences in informal care costs and decreased substantially when physical capacity was accounted for in the analyses; e.g., the mean difference in societal costs between sufficient and insufficient PA decreased from -€7,615 (95% CI [-11,404; -3,825]) to -€4,532 (95% CI [-7,930; -1,133]).

**Conclusion:**

Promoting PA throughout the lifespan as a means of promoting healthy aging and reducing dependency in old age could potentially provide economic benefits and help to mitigate the economic consequences of an aging population with increasing health and long-term care needs. Future longitudinal studies should attempt to disentangle the mediating and confounding role of physical capacity and health status in the association between PA and costs.

## Background

 Worldwide, but especially in western, industrialized countries such as Germany, the population is aging rapidly, and will continue to do so in the future [1]. This trend poses a challenge to the sustainability of social health insurance and pension systems (such as in Germany), which are heavily dependent on income-related contributions from the shrinking working population relative to the growing number of older people. *Healthy aging* as “the process of developing and maintaining the functional ability that enables wellbeing in older age” ([2], page 2149) has been recognized as a public health priority and can potentially buffer the costs in older age groups. This has led to an increased interest in addressing the problems of an age-related decline in health and physical capacity (e.g., prevent frailty), and promoting participation and well-being through lifestyle interventions (eg, the PromeTheus project [3]). Physical activity (PA) is an important contributor to healthy aging as it prevents or slows down the decline in health and physical function, and improves psychological well-being [4–6]. For adults aged 65 years and older, the World Health Organization (WHO) recommends engaging in at least 150 min per week of moderate-intensity PA (or an equivalent combination of moderate-to-vigorous PA), which should include PA that emphasizes functional balance and strength on at least three days per week [7].

International population-based studies indicate a high economic burden due to insufficient PA [8, 9]. However, few studies have examined the association between PA and costs in a German setting. As different countries have different healthcare systems with varying reimbursement schemes (and therefore different costs), it is often difficult to generalize cost estimates from other countries or healthcare systems. Karl et al. found a cross-sectional association of device-assessed, but not self-reported, physical inactivity with higher healthcare costs in a region-specific population-based sample from Germany [10]. Furthermore, a recent study based on cross-sectional data from the German National Cohort (NAKO) found that self-reported insufficient PA versus sufficient PA was associated with higher healthcare and societal costs, particularly in the population aged 60 + years [11]. However, these two studies did not include participants older than 75 years, nor did they consider costs associated with formal or informal care. While formal care comprises paid care services, which in Germany are largely covered by the long-term care insurance (which is linked to the statutory health insurance), informal care (i.e., by relatives, friends, acquaintances, etc.) is often provided without a formal contract or payment. Thus, informal care costs arise from the opportunity costs (e.g., giving up paid employment or free-time to provide care). Given an increasing population of older and oldest-old people and high prevalence rates of (pre-)frailty [12], the demand for formal and informal care is expected to rise, resulting in higher societal costs [13, 14]. Hence, it is relevant to better understand the economic impact of factors such as PA that contribute to healthy aging and can mitigate care dependency.

Therefore, the current study aimed to examine the association between PA and healthcare and societal costs (including costs for formal and informal care) in a sample of very mild to moderately frail older adults (aged 70 + years).

## Methods

This manuscript was prepared in accordance with the adapted Consolidated Health Economic Evaluation Reporting Standards (CHEERS) for studies examining the economic burden of physical inactivity and other risk factors [15].

### Study design and sample

This cross-sectional study is a secondary analysis using person-level data from 385 individuals from the baseline examination of the PromeTheus multicenter randomized-controlled trial that aimed to evaluate a multifactorial, interdisciplinary intervention program to prevent functional and mobility decline in (pre-)frail community-dwelling older adults [3] (registered on March 11, 2021, German Clinical Trials Register, ID: DRKS00024638). Participants were recruited between May 2021 and November 2022 in the areas of Stuttgart, Heidelberg, and Ulm (Baden-Wuerttemberg, Germany); either via general practitioners or directly via flyers in magazines, local newspapers, and personalized letters to members of the ‘Allgemeine Ortskrankenkasse’ (AOK, one of the largest statutory health insurance companies in Germany). Persons were eligible for inclusion if they were members of the AOK Baden-Württemberg, were aged 70 years or older, had very mild to moderate frailty (Clinical Frailty Scale [16] score 4–6), lived at home or in assisted living (but not in a long-term care facility/nursing home), and were able to walk at least 10 m (but no more than 800 m) with or without a walking aid. Reasons for exclusion were cognitive impairment, insufficient German language skills, limited visual acuity, or certain medical conditions (e.g., heart failure, recent stroke, Parkinson’s disease, current cancer treatment, severe lung disease, or multiple sclerosis). Detailed eligibility criteria and an analysis of the recruitment strategies are reported elsewhere [3, 17].

### Costs

Costs were calculated based on self-reported resource use collected in a face-to-face interview setting with the questionnaire for the use of medical and non-medical services in old age (FIMA) [18]. Participants were asked about their utilization of outpatient services (number of visits to the general practitioner, various specialist physicians and therapists), inpatient and rehabilitation services (e.g., number of days in hospital or rehabilitation clinic, day clinic), medications (frequency and dose), formal and informal care (number of days and average hours of receiving support by a mobile nursing service, payed household help, family/friends/neighbors, and number of days in daycare or short-term nursing care), and medical devices bought in the last 6 months prior to the baseline assessment (varying time horizons of the original FIMA were adapted accordingly). Resource use was monetarily valued using published standardized unit costs in euros [19], inflated to the year 2022 [20]; medications were monetarily valued by pharmacy retail prices [21]. The unit costs (hourly rate) based on the opportunity costs for paid work (average gross labor costs) were taken for the monetary valuation of informal care [19]. Costs were summarized as total 6-month societal costs (all cost categories) and total healthcare costs (excluding informal care costs).

### Physical activity

In the PromeTheus trial, self-reported PA was also obtained in a face-to-face interview using the German Physical Activity Questionnaire for middle-aged and older adults (German-PAQ-50+) [22]. The German-PAQ-50 + asks individuals about the time spent on several activities in the domains of housework, gardening, free time, sports, and occupation in a typical week within the last month. Each activity is assigned a specific metabolic equivalent (MET) [23], which is used to weight the energy expenditure of the activity against the energy expenditure while sitting at rest (= 1 MET). The total and domain-specific weekly energy expenditure is measured in MET-hours per week (MET-h/wk), calculated by multiplying the time spent on a particular activity in hours by its corresponding MET value. For the analyses in this study, PA intensity categories were built based on moderate- to vigorous-intensity activities only (≥ 3 MET [24]) to reflect (non-)adherence to the WHO PA recommendation of at least 150 min of moderate to vigorous PA per week (equivalent to ≥ 7.5 MET-h/wk) [7]: insufficient (< 7.5 MET-h/wk), moderate (7.5 to < 15 MET-h/wk), and high (≥ 15 MET-h/wk).

### Covariates

Covariates considered for adjustment of the analyses were socio-demographic characteristics (age [in years], gender [male; female], educational degree [low; intermediate; high; highest; no degree], family status [married; unmarried; divorced; widowed]), and physical capacity (Short Physical Performance Battery, SPPB, ranging from 0 [worst] to 12 [best] [25]). Comorbidities such as myocardial infarction, congestive heart failure, peripheral arterial disease, stroke, chronic lung disease, diabetes mellitus, cancer, etc. were assumed to be mediators in the association and thus were not included as covariates in this study.

### Statistical analysis

Descriptive statistics were used to summarize the sample’s sociodemographic characteristics and health and functional status. Adjusted mean healthcare and societal costs for different PA levels (sufficient vs. insufficient; insufficient vs. moderate vs. high) were estimated from generalized linear models (GLMs) with a Gamma distribution and log-link function. This type of GLMs has been found to precisely estimate population means of skewed cost data, even with relatively small sample sizes, while avoiding issues related to back-transformation when using transformed scales (e.g., the natural logarithm) [26, 27]. When analyzing the difference between PA levels by cost category (outpatient, inpatient, formal and informal care, medications and medical devices), two-part models were calculated when there were excess zeros in the dependent variable [28]. The skewed and outlier-influenced cost data were additionally addressed by estimating quantile regression models (also known as generalized median regressions) to compare adjusted median costs (the 50% quantile) between PA categories. All models were adjusted in two steps. First, only age, gender, educational degree, and family status were included as covariates (Model 1). Second, the models were additionally adjusted for physical capacity (Model 2) to examine its potential confounding effect on the association (e.g., pre-existing limitations in physical capacity could be an expression of poor health and be associated with both reduced PA and high health-related costs).

95% confidence intervals (CI) based on robust standard errors were calculated and reported alongside the mean/median. There were no missing values in the variables of interest in this study. All analyses were conducted using STATA/SE 18.0 [StataCorp. 2023. Stata Statistical Software: Release 18. College Station, TX: StataCorp LLC].

**Table 1:** Sample characteristics of 385 community-dwelling very mild to moderately frail older adults participating in the PromeTheus randomized controlled trial

Age - mean (SE)	81.2 (0.3)
Female gender - n (%)	283 (73.5)
Family status - n (%)	
Married	118 (30.6)
Married, living separated	4 (1.0)
Unmarried	26 (6.8)
Divorced	38 (9.9)
Widowed	199 (51.7)
Educational degree - n (%)	
Low^a^	244 (63.4)
Intermediate^b^	84 (21.8)
High^c^	14 (3.6)
Highest^d^	31 (8.1)
No degree	12 (3.1)
Vocational degree - n (%)	
Vocational school^e^	227 (59.0)
Master school/technical college^f^	41 (10.6)
Engineering college etc.^g^	1 (0.3)
University of applied sciences^h^	12 (3.1)
University	21 (5.5)
No vocational degree	83 (21.6)
Living situation - n (%)	
Private household	261 (67.8)
Assisted living	124 (32.2)
Care degree^i^ - n (%)	
None	236 (61.5)
Level 1	53 (13.8)
Level 2	78 (20.3)
Level 3	17 (4.4)
Use of an assistive medical device - n (%)	273 (70.9)
Fall history in past 6 months - n (%)	142 (36.9)
Body mass index - mean (SE)	29.4 (0.3)
Clinical frailty scale (range: 1 to 9) [16] - mean (SE)	4.4 (0.05)
EQ-5D-5 L index (range: -0.661 to 1) [29] - mean (SE)	0.74 (0.01)
EQ-VAS (range: 0 to 100) - mean (SE)	59.5 (0.9)
Short FES-I (range: 7 to 28) - mean (SE)	12.6 (0.2)
MET-hours/week (German-PAQ-50+) - mean (SE)	69.6 (2.1)
SPPB score (range: 0 to 12) - mean (SE)	6.5 (0.1)
Healthcare costs (6 months) - mean (SE)	5,342 (470)
Societal costs (6 months) - mean (SE)	9,940 (789)

^a^Hauptschul-/Volksschulabschluss, ^b^Realschulabschluss/Mittlere Reife, ^c^Fachabitur/Fachhochschulreife, ^d^Abitur, ^e^Berufsschule; ^f^Fachschule/Techniker-/Meisterschule etc.; ^g^Ingenieur-Schule/Polytechnikum etc.; ^h^Fachhochschule; ^i^German, Pflegegrad“

*German-PAQ-50+* German Physical activity questionnaire 50+ [22], *MET* metabolic equivalent, *Short FES-I* Short Falls Efficacy Scale-International [30], *SPPB* Short Physical Performance Battery [25]

## Results

Table 1 gives an overview of the sample characteristics. The mean age was 81.2 years and the majority (73.5%) were female. About half of the sample were widowed and another 30.6% were married. The majority had a low (63.4%) to intermediate (21.8%) educational degree, which was also reflected in the vocational degree, where the majority had either graduated from vocational school (59.0%) or had no vocational degree (21.6%). Most participants were living at home (67.8%) and had either no or the lowest care degree (75.3%, no to minor impairment of independence), while a smaller percentage were living in an assisted living facility (32.2%) and had care level 2 or 3 (24.7%, severe impairment of independence). On average, participants were moderately concerned about falling (Short FES-I = 12.6, standard error (SE) = 0.2) and had a mean SPPB score of 6.5 (SE = 0.1), indicating poor to moderate physical capacity. The mean Clinical Frailty Scale score of 4.4 (SE = 0.05) indicated that the sample had very mild to mild frailty.

**Figure 1:**
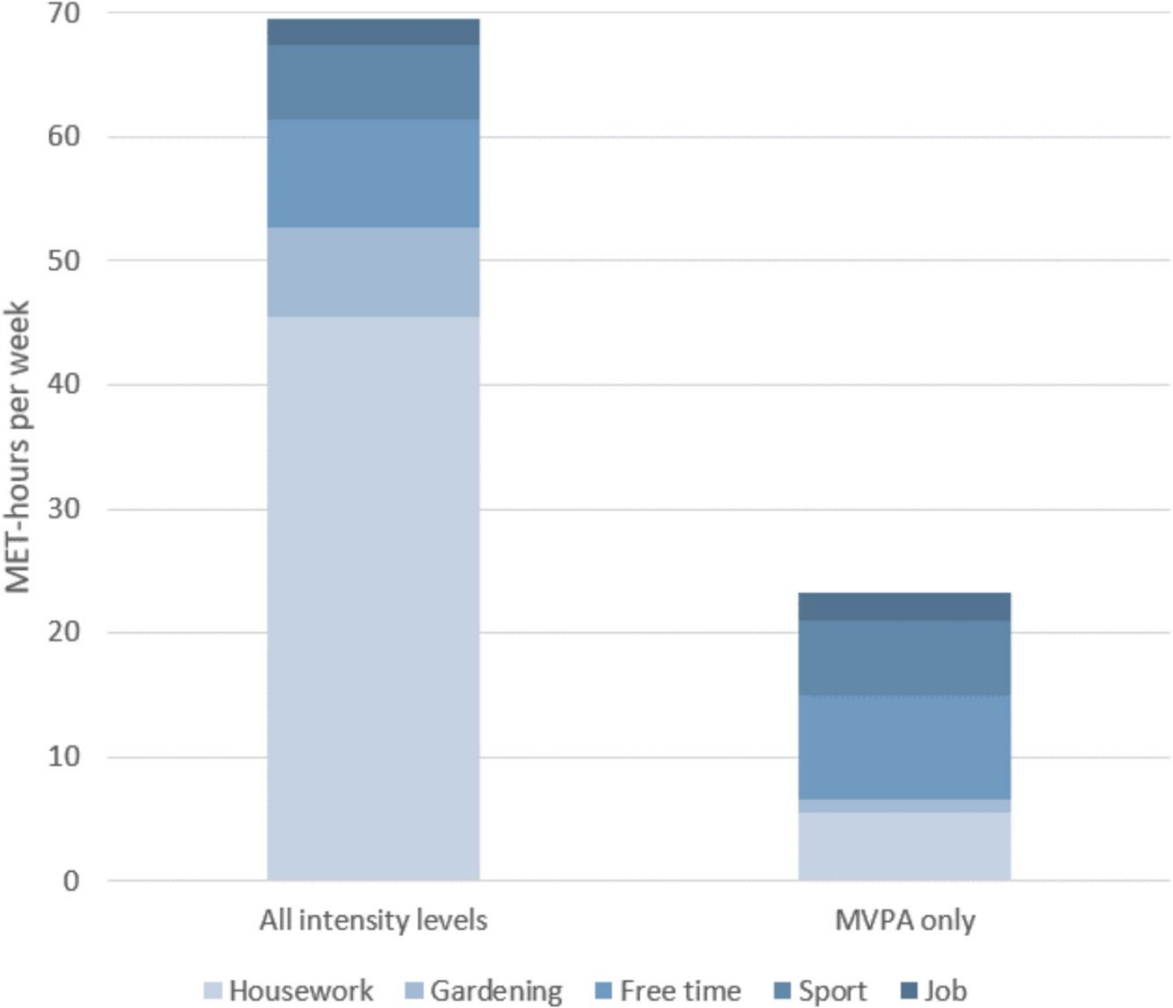
Weekly energy expenditure of community-dwelling older adults with very mild to moderate frailty (*N*=385) by German-PAQ-50+ domains. MVPA, moderate to vigorous physical activity; MET, metabolic equivalent.

The participants’ mean activity level was 69.6 MET-h/wk, accumulated in the domains of housework, gardening, free time, sports, and job (Fig. 1). A large proportion of the energy expenditure (40.0 MET-h/wk) was accumulated through light-intensity housework activities. When considering only moderate-to-vigorous activities (those that count towards achieving the WHO PA recommendations and were used for the PA classification in this study), the total activity level was considerably lower (23.2 MET-h/wk), but still exceeded the WHO PA recommendations for weekly aerobic PA (≥ 150 min moderate-intensity PA ≙ ≥ 7.5 MET-h/wk) [7].

Adjusting for sociodemographic characteristics only (Model 1), sufficiently active individuals (*n* = 294, 76.4% of the sample) had considerably lower mean and median healthcare (-€2649, 95% CI [-4,993; -304] and -€1,396, 95% CI [-2,407; -385], respectively) and societal costs (-€7,615, 95% CI [-11,404; -3,825] and -€6,595, 95% CI [-9,616; -3,574]; Table 2). With additional adjustment for physical capacity (Model 2), the difference between groups became smaller, but the point estimates still indicated lower costs in individuals meeting the PA recommendations, especially from a societal cost perspective (mean Δ: -€4,532, 95% CI [-7,930; -1,133] and median Δ: -€3,875, 95% CI [-6,769; -980]).

**Table 2:** Six-month mean/median costs (2022 euros) for sufficiently (*n* = 294) vs. insufficiently (*n* = 91) active community-dwelling very mild to moderately frail older adults participating in the PromeTheus randomized controlled trial

	Mean (95% CI)	Median (95% CI)
Healthcare costs		
Model 1		
Sufficient PA	4,713 (3,763; 5,663)	2,203 (1,789; 2,618)
Insufficient PA	7,362 (5,178; 9,545)	3,599 (2,626; 4,572)
Δ	-2,649 (-4,993; -304)	-1,396 (-2,407; -385)
Model 2		
Sufficient PA	5,017 (3,929; 6,105)	2,595 (2,182; 3,007)
Insufficient PA	6,412 (4,623; 8,202)	3,117 (2,278; 3,956)
Δ	-1,395 (-3,434; 645)	-522 (-1,431; 387)
**Societal costs**		
Model 1		
Sufficient PA	8,032 (6,465; 9,599)	4,266 (3,400; 5,133)
Insufficient PA	15,647 (12,198; 19,096)	10,861 (7,898; 13,825)
Δ	-7,615 (-11,404; -3,825)	-6,595 (-9,616; -3,574)
Model 2		
Sufficient PA	8,550 (6,931; 10,170)	4,930 (3,925; 5,936)
Insufficient PA	13,082 (10,195; 15,969)	8,805 (6,106; 11,504)
Δ	-4,532 (-7,930; -1,133)	-3,875 (-6,769; -980)

Model 1: adjusted for age, gender, educational degree, and family status. Model 2: adjusted for the covariates in Model 1 plus physical capacity (Short Physical Performance Battery, SPPB)

*PA* physical activity, *CI* confidence interval, Δ delta (difference)

Looking at different cost categories, the mean differences between the sufficient and insufficient PA groups were highest for informal care (-€4,950, 95% CI [-7,698; -2,201]), followed by inpatient care (-€2,174, 95% CI [-4,662; 313]) and formal care (-€533, 95% CI [-888; -179]); but again, the cost differences decreased considerably after frailty status was included in the models (Table 3).

**Table 3:** Six-month mean costs (2022 euros) by cost category for sufficiently vs. insufficiently active community-dwelling very mild to moderately frail older adults participating in the PromeTheus randomized controlled trial

	Inpatient	Outpatient	Medications	Formal care	Informal care	Medical aids/assistive devices
	Mean (95% CI)	Mean (95% CI)	Mean (95% CI)	Mean (95% CI)	Mean (95% CI)	Mean (95% CI)
Model 1						
Sufficient PA	2,152 (1,304; 3,000)	910 (822; 997)	763 (581; 945)	752 (606; 898)	3,281 (2,356; 4,206)	83 (50; 116)
Insufficient PA	4,326 (1,991; 6,662)	1,016 (808; 1,224)	774 (591; 957)	1,285 (962; 1,608)	8,231 (5,583; 10,878)	176 (50; 302)
Δ	-2,174 (-4,662; 313)	-106 (-324; 112)	-11 (-210; 187)	-533 (-888; -179)	-4,950 (-7,698; -2,201)	-93 (-222; 36)
Model 2						
Sufficient PA	2,371 (1,337; 3,405)	924 (835; 1,014)	786 (602; 970)	814 (656; 971)	3,563 (2,679; 4,447)	88 (52; 124)
Insufficient PA	3,657 (1,771; 5,544)	964 (767; 1,161)	686 (544; 829)	1,075 (797; 1,354)	6,488 (4,413; 8,564)	149 (37; 262)
Δ	-1,286 (-3,433; 860)	-40 (-251; 171)	100 (-110; 309)	-261 (-586; 63)	-2,926 (-5,201; -650)	-61 (-181; 59)

Model 1: adjusted for age, gender, educational degree, and family status. Model 2: adjusted for the covariates in Model 1 plus physical capacity (Short Physical Performance Battery, SPPB)

*PA* physical activity, *CI* confidence interval; Δ, delta (difference)

When the sufficiently active group was further divided into moderate (*n* = 88, 22.9%) and high PA (*n* = 206, 53.5%), the high PA group consistently had the lowest mean and median costs (Table 4). After controlling for physical capacity, the high PA group had lower mean healthcare costs (-€1,688, 95% CI [-3,727; 352]) and societal costs (-€5,033, 95% CI [-8,478; -1,588]) compared to the group with insufficient PA.

**Table 4:** Six-month mean/median costs (2022 euros) for PA intensity levels (insufficient [*n* = 91], moderate [*n* = 88], high [*n* = 206]) of community-dwelling very mild to moderately frail older adults participating in the PromeTheus randomized controlled trial

	Mean (95% CI)	Δ (95% CI)	Median (95% CI)	Δ (95% CI)
Healthcare costs				
Model 1				
Insufficient PA	7,382 (5,219; 9,544)	ref.	3,585 (2,633; 4,538)	ref.
Moderate PA	5,743 (3,659; 7,826)	-1,639 (-4,516; 1,238)	2,812 (2,188; 3,436)	-773 (-1,876; 330)
High PA	4,274 (3,431; 5,116)	-3,108 (-5,438; -778)	2,021 (1,562; 2,479)	-1,564 (-2,579; -550)
Model 2				
Insufficient PA	6,437 (4,640; 8,233)	ref.	3,356 (2,544; 4,168)	ref.
Moderate PA	5,511 (3,458; 7,565)	-925 (-3,543; 1,693)	2,745 (2,001; 3,489)	-611 (-1,689; 467)
High PA	4,749 (3,765; 5,734)	-1,688 (-3,727; 352)	2,479 (2,026; 2,932)	-877 (-1,786; 32)
**Societal costs**				
Model 1				
Insufficient PA	15,757 (12,290; 19,224)	ref.	10,894 (7,978; 13,810)	ref.
Moderate PA	9,520 (6,704; 12,337)	-6,237 (-10,656; -1,817)	4,873 (3,538; 6,208)	-6,021 (-9,161; -2,882)
High PA	7,424 (5,724; 9,124)	-8,333 (-12,183; -4,483)	3,924 (2,960; 4,888)	-6,970 (-9,977; -3,964)
Model 2				
Insufficient PA	13,137 (10,234; 16,040)	ref.	9,009 (6,293; 11,724)	ref.
Moderate PA	9,456 (6,548; 12,364)	-3,681 (-7,807; 446)	5,023 (3,765; 6,281)	-3,985 (-6,984; -987)
High PA	8,104 (6,459; 9,749)	-5,033 (-8,478; -1,588)	4,878 (3,793; 5,962)	-4,131 (-7,061; -1,201)

Model 1: adjusted for age, gender, educational degree, and family status. Model 2: adjusted for the covariates in Model 1 plus physical capacity (Short Physical Performance Battery, SPPB)

*PA* physical activity, *CI* confidence interval, Δ delta (difference)

## Discussion

In this cross-sectional analysis based on a sample of very mild to moderately frail adults aged 70 years and older, engaging in a sufficient level of PA was associated with lower costs, indicating that maintaining PA in old age may potentially be economically beneficial. The cost difference between PA intensity groups was largely driven by differences in informal care costs and decreased substantially when physical capacity was taken into account (from -€7,615, 95% CI [-11,404; -3,825] to -€4,532, 95% CI [-7,930; -1,133]). This highlights the link between PA and physical capacity, the causal or temporal relationship of which, however, cannot be clearly differentiated in this cross-sectional study: Physical capacity could act both as a mediator and a confounder. A confounding effect would mean that increasing PA (in older age) is not always a matter of choice or individual motivation, but also depends on physical capacity. Therefore, simply recommending more physical activity to older individuals (assuming that physical capacity solely acts as mediator), particularly to those who are already frail and physically limited, would not be sufficient. Instead, it may be necessary to first address preconditions for physical activity, such as physical capacity and performance, as well as treating musculoskeletal pain, improving nutritional status, and providing adequate medication. Support for the mediating role of physical capacity is provided by one of the few longitudinal studies in the field: The authors found that being consistently active during the transition from mid-age to older age was associated with the lowest costs [31], emphasizing the importance of maintaining the capacity to be active throughout the life course to reduce dependency in old age. This can reduce the need for health-related resources, especially for (in)formal care or support services, and thus also reduce costs. This is especially relevant in view of the growing population of older people and the projected shortage of formal and informal care [13, 32].

The current study’s findings align with previous studies that examined the association between PA and costs in an older population. For example, in a sample of community-dwelling older adults from Japan, Yang et al. report that medical care costs were lower with higher PA, even after controlling for chronic conditions and physical performance [33]. Liu-Ambrose et al. found a negative association between PA and costs related to health resource utilization among community-dwelling adults aged 65 years and older from Canada, but also emphasized the importance of accounting for overall health in the analyses, as this had the largest effect on costs [34]. Similarly, controlling for physical capacity considerably reduced the cost difference between PA groups in the current study.

In a population-based sample aged 20 to 74 years from the German NAKO study, the cost difference between sufficiently and insufficiently active people was especially pronounced in the 60 + age group [11]. The results of the current study point to an even larger difference beyond the age of 70 in terms of direct healthcare costs, but especially when the costs of informal care were taken into account, which were not surveyed in the NAKO and therefore not included in the analyses. The (informal) care need becomes particularly apparent with increasing age and is linked to the (decline in) physical capacity and the level of independence. PA can help to slow down the loss of function that leads to care dependency [4–6]. Vice versa, physically less active or inactive people are likely to have a lower level of functioning, which is associated with a higher need for informal and formal care (which have also found to be complementary [35]). Whereas in the NAKO-based analysis, increasing PA to an energy consumption equivalent to ≥ 300 min spent in moderate activity per week (= high PA) did not result in even lower costs compared to moderately active people, in the current study, the highly active group always had the numerically lowest mean or median costs. However, the results may not be directly comparable, as the individual items and PA domains of the Global Physical Activity Questionnaire (GPAQ) [36] used in the NAKO study differ from those of the German PAQ-50+. For example, the GPAQ has a separate dimension with questions about activities related to travelling to and from places, which was not specifically asked in the German-PAQ 50+. In addition, sports, fitness, and recreational activities are summarized under the dimension leisure in the GPAQ, while the German-PAQ 50 + has separate dimensions for gardening, leisure, and sports with more detailed questions on specific activities.

Compared to the previous studies, the current study widened the cost perspective by including informal care costs, the cost category in which cost differences between PA groups were the largest. Thereby, the current study highlighted the wider societal consequences of insufficient PA beyond the healthcare system.

### Limitations

Some limitations must be highlighted. A large proportion (54%) of the sample was categorized as ‘highly active’, which points to the common problem of over-reporting PA in self-report assessments [37, 38]. The absolute activity levels were unrealistically high, requiring an interpretation with great caution. However, we assume that the German-PAQ 50 + still captures the differences within our sample, which is the main purpose of our analysis. Similarly, self-reported health-related resource use may have led to an underestimation of costs as it is prone to recall bias (especially in an older population that often uses multiple healthcare services) [39]. There might also have been an impact of the COVID-19 pandemic on resource use (e.g., avoidance of non-essential physician visits) or physical activity patterns. Therefore, future studies are needed that base their analyses on more objectively measured PA and cost data. Moreover, the analyses were based on a rather small sample that is not representative of the population aged 70 + in Germany, as participants had to fulfill certain eligibility criteria to be included in the trial [3]. Finally, the cross-sectional design does not allow for causal conclusions. Thus, future studies should examine the economic consequences of increasing PA levels in a longitudinal design, also attempting to disentangle the mediating and confounding role of physical capacity and general health status.

## Conclusions

In a sample of very mild to moderately frail adults aged 70 years and older, obtaining sufficient PA levels was associated with lower societal and healthcare costs. The cost difference between PA intensity groups was particularly pronounced for informal care costs, and its magnitude depended on whether physical capacity was controlled for. Promoting PA across the life course to reduce dependency in old age may prove to be economically relevant in light of an aging population with increasing health and long-term care needs.

## Data Availability

The datasets generated and/or analyzed during the current study are not publicly available due to ethical and confidentiality concerns but are available from the corresponding author upon reasonable request.

## Abbreviations

German-PAQ-50+: German Physical Activity Questionnaire for middle-aged and older adults
GPAQ: Global Physical Activity Questionnaire
MET: metabolic equivalent
MET-h/wk: MET-hours per week
NAKO: German National Cohort
PA: Physical activity
Short FES-I: Short Falls Efficacy Scale-International
SPPB: Short Physical Performance Battery
